# *Gratiola officinalis* Alcoholic Extract Targets Warburg Effect, Apoptosis and Cell Cycle Progression in Colorectal Cancer Cell Lines

**DOI:** 10.3390/ijms26052220

**Published:** 2025-02-28

**Authors:** Stefano Bianchini, Federica Bovio, Stefano Negri, Flavia Guzzo, Matilde Forcella, Paola Fusi

**Affiliations:** 1 Department of Biotechnology and Biosciences, University of Milano-Bicocca, Piazza della Scienza 2, 20126 Milano, Italy; stefano.bianchini@unimib.it (S.B.); federica.bovio@unimib.it (F.B.); 2 Department of Biotechnology, University of Verona, Strada Le Grazie 15, 37134 Verona, Italy; stefano.negri@univr.it (S.N.); flavia.guzzo@univr.it (F.G.); 3 National Biodiversity Future Center (NBFC), 90133 Palermo, Italy; 4 Integrated Models for Prevention and Protection in Environmental and Occupational Health (MISTRAL), Interuniversity Research Center, 25121 Brescia, Italy

**Keywords:** colorectal cancer, *Gratiola officinalis*, verbascoside, glycolysis, energy metabolism

## Abstract

Colorectal cancer (CRC) is the second deadliest cancer in the Western world. Increased body weight, a diet rich in red meat and alcohol, as well as a sedentary lifestyle, are all involved in sporadic CRC pathogenesis. Since current CRC therapies show several side effects, there is a need to find new and more effective therapeutic approaches, allowing conventional drug dosages and toxicity to be reduced. *Gratiola officinalis* alcoholic extract was characterized by LC-MS and its effect investigated on a healthy colon mucosa cell line and on different colorectal cancer cell lines. Cell viability, apoptosis and cell cycle progression were evaluated through flow cytometry; energy production and glycolysis were investigated using Seahorse technology, while cancer markers were analyzed through Western blotting. The untargeted metabolomics analysis of *G. officinalis* alcoholic extract revealed glycosides of different polyphenols and glycosides of cucurbitane-type triterpenes. This extract showed a stronger impact on CRC cell line viability compared to healthy colon cells. In the E705 CRC cell line, it induced cell apoptosis and caused the downregulation of glycolysis, inhibiting cell proliferation. On the other hand, SW480 CRC cells treated with *G. officinalis* extract showed G_2_/M cell cycle arrest. This work shows that *G. officinalis* extract can reduce glycolysis and promote cell cycle arrest in CRC cells, suggesting that *G. officinalis* could represent a novel player in the prevention and treatment of CRC.

## 1. Introduction

Common hedge hyssop (*Gratiola officinalis* L., Plantaginaceae) is one of the most versatile medicinal plants, although it is endowed with some toxic potential [1]. It has been used since the Middle Ages to treat liver diseases, visceral obstructions, skin diseases, menstrual disorders, gout and parasitic worms; it is also known to affect the nervous system [2]. Moreover, while its anti-inflammatory and sedative actions have been known for a long time, more recently, *G. officinalis* extracts have been shown to be endowed with antioxidant [3] and anticancer activity towards breast and cervical cancer cells [4]. In particular, Polukonova and coworkers also found that *G. officinalis* extracts induced apoptosis and necrosis in HCT-116 human colorectal cancer cells [5]. The flavonoid component in *G. officinalis* extracts was found to be involved in both antioxidant and anticancer activities. Bioflavonoids are known to exert a wide range of anticancer effects, like apoptosis induction, cell cycle arrest and angiogenesis inhibition [6].

With nearly one million newly diagnosed cases each year [7], colorectal cancer (CRC) is the second deadliest cancer in the Western world [8]. Hereditary forms, like familial adenomatous polyposis and hereditary non-polyposis colorectal cancer, account for only approximately 10–20% of all colorectal cancers, with most patients developing sporadic CRC [9]. In addition, sporadic CRC is highly represented among early-onset colorectal cancer patients, a group that has constantly increased over the past two decades, with over 80% of patients not showing any germline mutation [10,11]. In recent years, mortality in CRC patients under the age of 50 has shown an increasing trend, in contrast to the overall decreasing mortality rates among older adults [12].

Dietary factors, such as excessive red meat and alcohol intake, together with other factors like antibiotic assumption, increased bodyweight and a sedentary lifestyle, are involved in sporadic CRC pathogenesis; these factors make CRC a “Westernized” disease, with a high incidence in North America, Australia, New Zealand and Europe [8]. Moreover, the same dietary and environmental factors, disrupting colon epithelium homeostasis, often induce dysbiosis, a potential causal factor for early-onset CRC, especially in women [13,14].

On the other hand, protective factors associated with a decrease in the incidence of CRC include regular physical activity and a diet rich in fruits, vegetables, fish and fiber. An adequate intake of folate, calcium, vitamin D, vitamin B6 and magnesium has also been shown to play a significant role in CRC prevention [15,16].

Current CRC therapies depend on cancer stage: non metastatic CRC is treated with radiotherapy and/or chemotherapy, while for metastatic cancer, the treatment of choice is systemic therapy, including chemotherapy, targeted therapies and immunotherapy [17]. The latter aims at inhibiting epidermal growth factor receptor (EGFR) dimerization, using monoclonal antibodies against EGFR, such as cetuximab and panitumumab. However, these drugs are not effective in patients carrying *RAS*/*BRAF* mutations, whose prognosis is generally more unfavorable [18]. Moreover, even patients who are successfully treated with systemic therapy undergo the potentially serious side effects of these drugs and often develop a resistance to anti-EGFR therapies, which can be due to the tumor heterogeneity or to the multidrug treatment favoring resistance through epigenetic changes or microenvironmental factors [18]. Hence, there is a need for new and more effective therapies, which could also be used to reduce conventional drugs’ dosages and toxicity.

In recent years, many plant extracts and compounds have been investigated for their anticancer activity towards CRC; different kinds of molecules, ranging from flavonoids to alkaloids, have been found to be endowed with anticancer activity [19,20].

In this work, we report the anticancer activity of *G. officinalis* alcoholic extract in different CRC cell lines, after assessing the lack of toxic activity on healthy colorectal control cells. We also explore the molecular mechanisms responsible for this anticancer activity, showing that *G. officinalis* extract can induce G_2_/M cell cycle arrest or downregulate glycolysis, which is hyperactivated in cancer cells, thus inhibiting cell proliferation.

## 2. Results and Discussion

### 2.1. Phytochemical Characterization of Gratiola officinalis Extract

**Figure 1:**
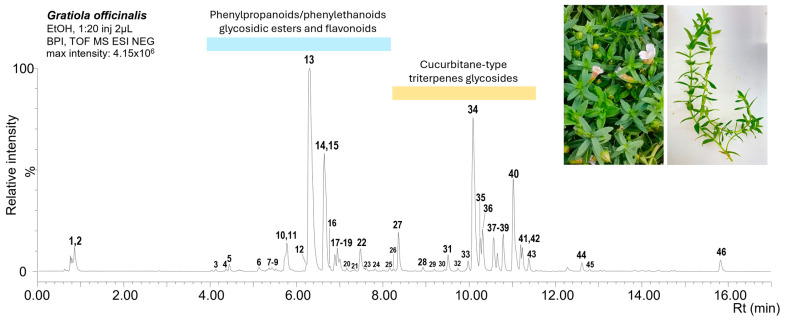
Base peak chromatogram of *G. officinalis* extract profiled by UPLC-ESI-HRMS in negative ionization mode. Numbers refer to metabolites listed in Table 1.

The untargeted metabolomics analysis of *G. officinalis* extract revealed a phytocomplex characterized by two main classes of metabolites: the glycosides of different polyphenols (phenylethanoids/phenylpropanoids esters and flavonoids) and the glycosides of cucurbitane-type triterpenes, eluting, respectively, in the medium-polar (4–8 min) and low-polar (8–13 min) range of the analysis (Figure 1).

Table 1 reports all the metabolites putatively identified with their LC-MS features (further information supporting the identification of the major metabolites detected is reported in Supplementary File S1).

The main metabolites from the polyphenol group were three glycosidic esters (peaks 13–15) featuring 2-(3,4-dihydroxyphenyl)ethanol (i.e., 3-hydroxytyrosol, phenylethanoid backbone) and caffeic acid (phenylpropanoid backbone) moieties: arenarioside/forsythoside B and verbascoside, the latter coeluting with samioside. These compounds were previously reported as the principal polyphenols of *G. officinalis* [21]. Further identifications were challenged by the high number of isomers observed in this metabolite class within the Lamiales. Thus, we tentatively identified the following compounds based on the information reported for species from other genera in the Plantaginaceae (e.g., Globularia, Plantago, Veronica) or phylogenetically related families: other glycosidic esters with 3-hydroxytyrosol and caffeic acid (echinacoside, isomers of echinacoside and verbascoside, acetyl-verbascoside) or with ferulic acid (leucosceptoside A) moietes; glycosidic esters with 2-(3-hydroxy-4-methoxyphenyl)ethanol and ferulic acid moieties (alyssonoside and martynoside); and 3-hydroxytyrosol glycosides (verbascoside and markhamioside A/peiioside B). Free caffeic acid and its conjugated forms with hexose sugars were also detected but at lower amounts. Flavonoids were mainly represented by the glycosides of the flavone apigenin (apigenin-C-pentoside-C-hexoside isomers) or its derivatives (e.g., methoxyapigenin). Besides their main antioxidant and anti-inflammatory activities, polyphenols can also modulate the intestinal microbiota. When they reach the distal part of the intestine, they are hydrolyzed and metabolized by intestinal enzymes and the gut microbiota. In particular, the gut microbiota can carry out demethylation and dehydroxylation reactions; conjugates are hydrolyzed releasing aglycones, which are absorbed by the colon and carried to the liver where they undergo further transformation [22]. Unabsorbed metabolites can modulate gut microbiota, exerting both a prebiotic effect, stimulating the growth of Lactobacilli, Bifidobacteria, etc., and an antimicrobial action against dpathogenic bacteria [23].

The occurrence of cucurbitane-type tetracyclic triterpenes in *G. officinalis* was previously reported [24,25], supporting our putative identifications of different glycosides (mainly 2-O-glucosides) of cucurbitacins B, E, I and S (also detectable, yet at lower levels, in their free form); the main peak from this group was attributed to cucurbitacin E-2-O-glucoside (also known as gratiotoxin/elaterinide, peak 34). Other cucurbitane-type triterpenes reported by the authors and putatively identified were those derived from gratiogenin, which, differently from cucurbitacins, seem to be exclusive of *G. officinalis* and harbor a furan ring in their structures; these include mainly 3-O-glucosyl-25-O-glucosyl-gratiogenin (gratioside) and its derivatives (e.g., peak 40), together with hydroxylated and 16-hydroxygratiogenin glycosides. At lower levels, triterpene acids, such as lupane-type pentacyclic triterpene betulinic acid, were also detected.

Concerning the bioactivities reported for the main phytochemicals detected in the extract, samioside and verbascoside are used in traditional herbal therapies and have been shown, in recent years, to be endowed with antiprotozoan activity [26,27]. Verbascoside anticancer activity has been studied by different authors in breast cancer [28]. A significant anti-metastatic and anti-invasion activity has been found in CRC HT-29 cultured cells [29] and a potential role has been suggested as an adjuvant treatment to decrease the resistance of CRC cells to 5-Fluorouracil, possibly by targeting the PI3K/AKT pathway [30]. Other components with a previously demonstrated anticancer effect towards CRC include caffeic acid phenethyesther [31], ferulic acid [32] and apigenin [33]. Among triterpenoids, pronounced antitumor and pro-apoptotic activity was reported for *G. officinalis* cucurbitacins in human kidney cancer Caki-1 and Sn12c cells [34].

**Table 1:** LC-MS features of the metabolites identified in *G. officinalis* extract. ID numbers refer to peaks indicated in Figure 1; * = metabolites identified by comparison with the authentic standard; and † = fragments observed in positive ionization. Abbreviations: PP, phenylpropanoid; PE, phenylethanoid. Further information supporting the putative identifications of these metabolites is reported in Supplementary File S1.

ID	Rt (min)	Putative Identification	Molecular Class	Formula	UV–Vis., λ Max. (nm)	ESI—Molecular Ion	ESI—Theoretical *m*/*z*	ESI—Experimental *m*/*z*	ESI—Δppm	ESI—Confirming Fragments	Reference
1	0.84	Tetra-hexose	Sugars	C_24_H_42_O_21_		[M+HCOOH−H^+^]^−^	711.2195	711.2200	0.65	665.2137; 383.1197; 341.1084; 179.0578; 221.0678; 485.1530	[35]
2	0.86	Di-hexose	Sugars	C_12_H_22_O_11_		[M+HCOOH−H^+^]^−^	387.1139	387.1138	−0.22	341.1084; 215.0318; 179.0578; 161.0464; 119.0344; 101.0243; 89.0222	Massbank
	2.82	Dihydroxybenzoic acid sulfohexoside	Benzoic acids	C_13_H_16_O_12_S		[M−H^+^]^−^	395.0284	395.0270	−3.60	241.002; 96.959; 153.019; 222.991; 138.970; 109.028; 222.9906; 80.9645	MetFrag
	3.03	Dihydroxybenzoic acid hexoside	Benzoic acids	C_13_H_16_O_9_		[M−H^+^]^−^	315.0716	315.0725	2.84	153.0184; 109.0278; 108.0214	Massbank
	4.04	Caffeoyl-hexose isomer 1	Hydroxycinnamic acids	C_15_H_18_O_9_		[M−H^+^]^−^	341.0873	341.0895	6.59	135.045; 179.036; 107.0492; 203.0383; 161.0257	Massbank
3	4.13	Verbasoside (decaffeoyl-verbascoside)	PE glycoside	C_20_H_30_O_12_		[M−H^+^]^−^	461.1659	461.1646	−2.80	135.045; 315.111; 297.099; 161.044; 113.024; 85.030	[36]
4	4.36	2,5-dihydroxy-p-benzenediacetic acid	Phenols	C_10_H_10_O_6_	257	[M−H^+^]^−^	225.0399	225.0390	−4.05	119.050; 207.0303; 163.0398; 107.049; 163.039;	[21]
5	4.43	Markhamioside A/Peiioside B	PE glycoside	C_25_H_38_O_16_	242, 324	[M−H^+^]^−^	593.2082	593.2104	3.79	161.0231; 447.1492; 461.1646; 135.0449; 429.1376; 153.0564	
	4.64	Caffeic acid glycoside	Hydroxycinnamic acids	C_19_H_26_O_12_		[M−H^+^]^−^	445.1346	445.1345	−0.21	161.023; 133.028; 179.033; 135.045; 153.0564	MetFrag
	4.68	Caffeoyl-hexose isomer 2	Hydroxycinnamic acids	C_15_H_18_O_9_	243, 324	[M−H^+^]^−^	341.0873	341.0858	−4.26	179.036; 135.047; 161.023; 221.043; 251.056; 281.067	[37]
6	5.13	UI		C_26_H_30_O_15_	239, 291, 327	[M−H^+^]^−^	581.1506	581.1526	3.38	269.0665; 239.0565; 209.0468	
	5.31	Caffeic acid *	Hydroxycinnamic acids	C_9_H_8_O_4_		[M−H^+^]^−^	179.0344	179.0359	8.20	135.0430; 133.0288	
	5.36	Apigenin-C-di-hexoside	Flavone glycosides	C_27_H_30_O_15_	255, 342	[M−H^+^]^−^	593.1506	593.1506	−0.06	353.0671; 383.0757; 473.1095; 297.0777; 503.1209; 325.0717	[38]
7	5.38	Tuberonic acid-O-hexoside	Jasmonates	C_18_H_28_O_9_		[M−H^+^]^−^	387.1655	387.1650	−1.29	163.1130; 369.0599; 119.0344; 207.104	[39]
8	5.43	Luteolin-C-pentoside-C-hexoside	Flavone glycosides	C_26_H_28_O_15_	251, 337	[M−H^+^]^−^	579.1350	579.1361	1.92	399.0723; 519.1184; 489.1015; 459.1009; 369.0599	[40]
9	5.52	UI with caffeoyl moiety		C_34_H_44_O_20_	247, 330	[M−H^+^]^−^	771.2348	771.2356	1.09	161.024; 179.034; 753.2252; 135.0449; 591.194; 661.199; 743.2308; 593.2155	
10	5.64	Echinacoside *	PP/PE ester glycoside	C_35_H_46_O_20_	271, 331	[M−H^+^]^−^	785.2504	785.2513	1.11	161.024; 623.219; 785.252	
11	5.69	Apigenin-C-pentoside-C-hexoside isomer 1	Flavone glycosides	C_26_H_28_O_14_		[M−H^+^]^−^	563.1401	563.1398	−0.42	353.077; 383.078; 297.0777; 443.0940; 473.1140; 325.0717	[38]
	5.91	Apigenin-C-pentoside-C-hexoside isomer 2	Flavone glycosides	C_26_H_28_O_14_		[M−H^+^]^−^	563.1401	563.1401	0.05	353.077; 383.078; 297.0777; 443.0940; 473.1140; 325.0717	[38]
	6.15	Echinacoside isomer	PP/PE ester glycoside	C_35_H_46_O_20_		[M−H^+^]^−^	785.2504	785.2544	5.09	179.0359; 623.2093; 161.024; 179.035; 461.1558; 135.0449;	[41]
12	6.23	UI with caffeoyl moiety		C_34_H_42_O_20_		[M−H^+^]^−^	769.2191	769.2234	5.58	179.0359; 135.0449; 161.0231; 149.0239; 619.1891	
13	6.31	Arenarioside/Forsythoside B	PP/PE ester glycoside	C_34_H_44_O_19_	245, 329	[M−H^+^]^−^	755.2398	755.2416	2.26	161.0231; 593.2104; 133.0282; 179.0359; 447.1492; 461.1690; 623.2042; 575.2020; 429.1418	[21]
14	6.65	Verbascoside *	PP/PE ester glycoside	C_29_H_36_O_15_	246, 329	[M−H^+^]^−^	623.1976	623.1988	1.88	161.026; 179.034; 461.168; 623.19	
15	6.68	Samioside	PP/PE ester glycoside	C_34_H_44_O_19_	244, 328	[M−H^+^]^−^	755.2398	755.2400	0.21	593.2104; 161.0257; 133.0292; 135.0449; 179.0332; 447.1492; 461.1690	[21]
	6.74	Isorhamnetin-O-hexoside	Flavonol glycosides	C_22_H_22_O_12_		[M−H^+^]^−^	477.1033	477.1027	−1.25	315.0527; 314.0435; 299.0231; 300.0274; 271.0276; 243.0321	
	6.75	Isorhamnetin-O-hexuronide	Flavonol glycosides	C_22_H_20_O_13_		[M−H^+^]^−^	491.0826	491.0818	−1.55	315.0527; 300.0274	
16	6.78	Alyssonoside	PP/PE ester glycoside	C_35_H_46_O_19_		[M−H^+^]^−^	769.2555	769.2588	4.30	593.2104; 175.0405; 160.0168; 193.0520; 135.0449; 447.1535; 575.2020; 461.1690; 315.1072	[42]
17	6.89	UI with caffeoyl moiety		C_36_H_46_O_20_	244, 329	[M−H^+^]^−^	797.2504	797.2537	4.13	179.0359; 161.0257; 135.0449; 619.1891; 635.2241	
18	6.94	Verbascoside isomer 1 (isoverbascoside)	PP/PE ester glycoside	C_29_H_36_O_15_	244, 326	[M−H^+^]^−^	623.1976	623.1990	2.27	161.024; 179.034; 461.164	[21]
19	7.00	Verbascoside isomer 2	PP/PE ester glycoside	C_29_H_36_O_15_	244, 326	[M−H^+^]^−^	623.1976	623.1990	2.27	161.024; 179.034; 461.164	[42]
20	7.17	UI with caffeoyl moiety		C_36_H_48_O_20_	247, 296, 327	[M−H^+^]^−^	799.2661	799.2662	0.18	161.0231; 637.2377; 608.1956; 179.0359; 133.0282	
	7.31	Leucosceptoside A	PP/PE ester glycoside	C_30_H_38_O_15_		[M−H^+^]^−^	637.2132	637.2119	−2.10	175.0405; 461.1690; 315.1108; 193.0491; 160.0168	[43,44]
21	7.35	Acetyl-verbascoside	PP/PE ester glycoside	C_31_H_38_O_16_		[M−H^+^]^−^	665.2082	665.2085	0.53	161.026; 179.034; 461.168; 623.1991	
22	7.48	Methoxyapigenin-O-hexuronide	Flavone glycosides	C_22_H_20_O_12_	250, 344	[M−H^+^]^−^	475.0876	475.0871	−1.08	284.0326; 299.0567	
23	7.62	Tuberonic acid-(caffeoyl)-O-hexoside	Jasmonates	C_27_H_34_O_12_		[M−H^+^]^−^	549.1972	549.1990	3.29	387.1690; 161.0257; 207.1039; 133.0305	
24	7.82	Dihydrodehydrodiconiferyl alcohol-9′-O-sulfate	Neolignan	C_20_H_24_O_9_S		[M−H^+^]^−^	439.1063	439.1071	1.88	96.9695; 269.0833; 314.1160; 299.9550; 79.9566	MetFrag; [45]
25	8.17	Martynoside	PP/PE ester glycoside	C_31_H_40_O_15_	246, 330	[M−H^+^]^−^	651.2289	651.2290	0.18	175.0405; 475.1821; 329.1217; 315.0527; 193.0520; 160.0168;	[42]
26	8.25	UI		C_23_H_40_O_9_		[M+HCOOH−H^+^]^−^	505.2649	505.2628	−4.10	459.2588; 417.2505; 399.2400; 161.0257	
27	8.37	Cucurbitacin I-2-O-glucoside	Cucurbitane-triterpene glycoside	C_36_H_52_O_12_	244	[M+HCOOH−H^+^]^−^	721.3435	721.3454	2.60	495.272; 513.286; 675.338	[46]
28	8.94	16-hydroxygratiogenin-3-O-(apiosyl)-glucoside-25-O-glucoside	Cucurbitane-triterpene glycoside	C_47_H_76_O_19_		[M+HCOOH−H^+^]^−^	989.4957	989.5006	4.97	943.494; 811.448; 649.396; 487.3421	[24]
29	9.19	C_30_H_46_O_6_-O-hexoside	Triterpenoid	C_36_H_56_O_11_		[M+HCOOH−H^+^]^−^	709.3799	709.3783	−2.25	663.3777; 501.3227; 163.0764	
30	9.43	UI		C_36_H_60_O_10_		[M+HCOOH−H^+^]^−^	697.4163	697.4187	3.47	nf	
31	9.52	C_30_H_50_O_5_-O-(pentosyl)-hexoside	Triterpenoid	C_41_H_68_O_14_		[M+HCOOH−H^+^]^−^	829.4585	829.4623	4.51	783.458; 651.413; 489.359	
32	9.74	Gratiogenin-3-O-(glucosyl)-glucoside-25-O-glucoside	Cucurbitane-triterpene glycoside	C_48_H_78_O_19_		[M+HCOOH−H^+^]^−^	1003.5114	1003.5098	−1.55	957.5112; 795.4572; 633.4042; 471.3524	[24]
33	9.98	Gratiogenin-3-O-(glucosyl)-glucoside-25-O-glucoside acetylated derivative	Cucurbitane-triterpene glycoside	C_51_H_80_O_22_		[M−H^+^]^−^	1043.5063	1043.5071	0.79	999.5259; 957.5112; 939.5005; 795.4572; 777.4499; 633.4042; 471.3524	
34	10.00	Cucurbitacin E-2-O-glucoside (elaterinide/gratiotoxin)	Cucurbitane-triterpene glycoside	C_38_H_54_O_13_	240	[M+HCOOH−H^+^]^−^	763.3541	763.3562	2.82	495.275; 657.329; 717.351; 615.3190; 699.3390; 537.2862; 477.2637	[46]
35	10.14	16-hydroxygratiogenin-3-O-(apiosyl)-glucoside	Cucurbitane-triterpene glycoside	C_41_H_66_O_14_		[M+HCOOH−H^+^]^−^	827.4429	827.4445	1.96	781.443; 649.398; 487.343	[24]
36	10.21	Cucurbitacin B-2-O-glucoside	Cucurbitane-triterpene glycoside	C_38_H_56_O_13_	252	[M+HCOOH−H^+^]^−^	765.3697	765.3716	2.42	497.283; 659.45; 701.353; 719.370; 557.3116	
37	10.46	Cucurbitacin S-2-O-glucoside	Cucurbitane-triterpene glycoside	C_36_H_52_O_11_	254	[M+HCOOH−H^+^]^−^	705.3486	705.3500	2.01	† ESI+: 349.1261]	[46]
38	10.55	Gratioside (gratiogenin-3-O-glucoside-25-O-glucoside)	Cucurbitane-triterpene glycoside	C_42_H_68_O_14_		[M+HCOOH−H^+^]^−^	841.4585	841.4603	2.14	795.4572; 633.4042; 471.3524	[24]
39	10.68	Gratiogenin-3-O-(apiosyl)-glucoside-25-O-glucoside	Cucurbitane-triterpene glycoside	C_47_H_76_O_18_		[M+HCOOH−H^+^]^−^	973.5008	973.5035	2.82	927.501; 795.4572; 633.4042; 471.3524	[24]
	10.93	Cucurbitacin I	Cucurbitane-triterpene glycoside	C_30_H_42_O_7_		[M+HCOOH−H^+^]^−^	559.2907	559.2898	−1.60	513.2865; 497.2931	
40	11.02	Gratioside acetylated derivative	Cucurbitane-triterpene glycoside	C_45_H_70_O_17_		[M−H^+^]^−^	881.4535	881.4554	2.21	777.449; 795.454; 837.468; 633.4042; 471.3436	
41	11.20	Gratiogenin-3-O-(apiosyl)-glucoside-25-O-glucoside acetylated derivative	Cucurbitane-triterpene glycoside	C_50_H_78_O_21_	252	[M−H^+^]^−^	1013.4957	1013.5010	5.22	777.448; 795.459; 909.494; 927.502; 969.515; 471.3524	
42	11.24	C_30_H_44_O_5_-di-O-hexoside	Triterpenoid	C_42_H_64_O_15_		[M+HCOOH−H^+^]^−^	853.4222	853.4229	0.87	807.4176; 645.3656; 483.3107	
43	11.39	Gratioside diacetylated derivative	Cucurbitane-triterpene glycoside	C_48_H_72_O_20_		[M−H^+^]^−^	967.4538	967.4559	2.12	879.477; 777.449; 759.436; 795.459; 837.472; 923.472; 819.4574; 633.404; 471.352	
	11.55	C_30_H_44_O_5_-di-O-hexoside acetylated derivative	Triterpenoid	C_38_H_70_O_23_		[M−H^+^]^−^	893.4229	893.4192	−4.19	849.4329; 807.4176; 789.4069; 645.3656; 644.3572; 163.0790	
44	12.63	Cucurbitacin E	Cucurbitane-triterpene glycoside	C_32_H_44_O_8_		[M+HCOOH−H^+^]^−^	601.3013	601.2994	−3.09	555.2984; 495.2776; 477.2682; 409.2032	[47]
	12.79	Cucurbitacin S	Cucurbitane-triterpene glycoside	C_30_H_42_O_6_		[M−H^+^]^−^	497.2903	497.2931	5.63	† ESI+: 481.2943; 463.2869; 317.2160]	[46]
45	12.81	Cucurbitacin B	Cucurbitane-triterpene glycoside	C_32_H_46_O_8_		[M+HCOOH−H^+^]^−^	603.3169	603.3190	3.47	557.3116; 479.2826; 497.2885; 539.3046; 339.1982; 163.0764	[47]
46	15.81	Betulinic acid *	Lupane-type triterpenic acid	C_30_H_48_O_3_		[M−H^+^]^−^	455.3525	455.3542	3.73	nf	[48]

### 2.2. G. officinalis Extract Triggers Apoptosis in Colorectal Cancer Cells, Without Affecting Healthy Colon Cells

MTT tests were performed on the colorectal cancer cell lines E705 and SW480; the former shows no hyperactivating mutations in *KRAS*, *NRAS* and *BRAF* and carries a silent mutation in the *PIK3CA* gene, while the latter carries a hyperactivating mutation in exon 2 of the *KRAS* gene. CCD 841 cells from healthy colon mucosa were used as controls.

**Figure 2:**
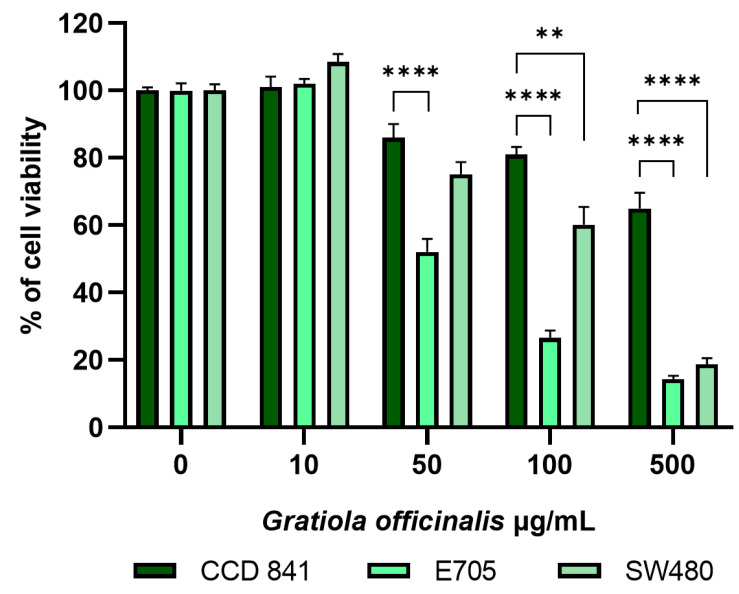
A MTT viability assay on the healthy colon mucosa CCD 841 cell line and colorectal cancer cell lines E705 and SW480. The cells were treated for 24 h with *G. officinalis* extract at concentrations between 0 and 500 μg/mL. Bars indicate the mean ± standard error (SE) of three individual experiments. Statistical significance: ** *p* < 0.01; **** *p* < 0.0001.

**Figure 3:**
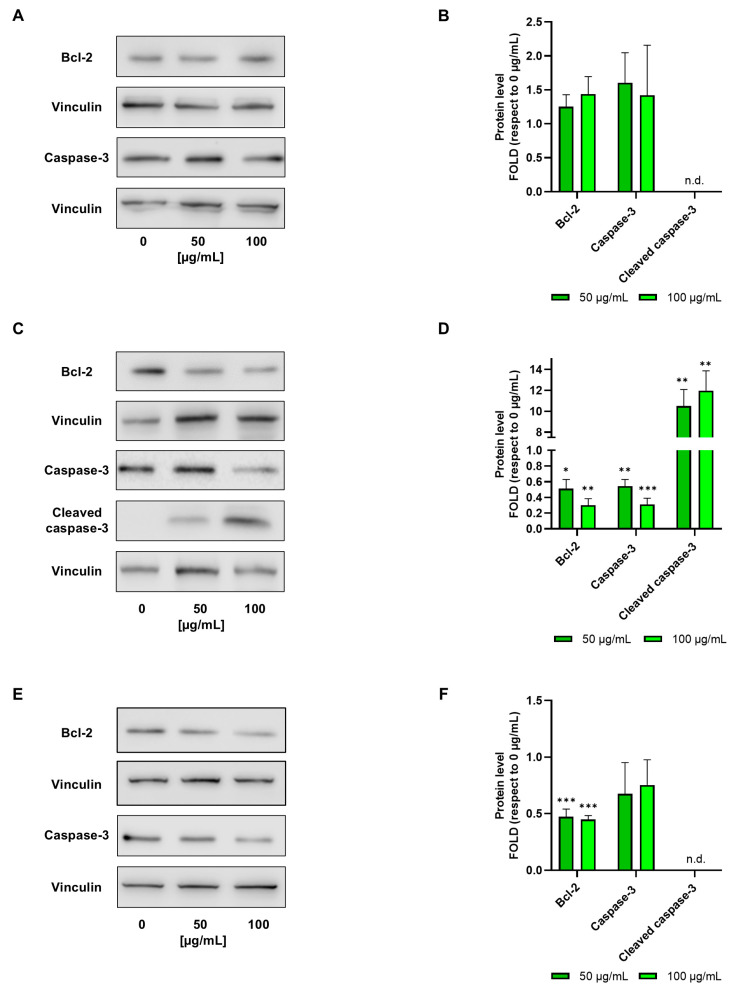
Western blot analysis. Representative Western blot analyses were performed on CCD 841 (**A**), E705 (**C**) and SW480 (**E**) cell lines that were untreated and treated for 24 h with 50 μg/mL and 100 μg/mL of *G. officinalis* extract. Protein extracts were separated on 12% acrylamide/bis-acrylamide SDS-PAGE and the nitrocellulose membranes were probed with anti-Bcl2 and anti-caspase-3 antibodies. Vinculin was used as a loading control. Densitometric analysis values are expressed as folds with respect to the control condition (0 μg/mL) and are presented as the mean ± standard error (SE) of three individual experiments (**B**,**D**,**F**). Statistical significance: * *p* < 0.05; ** *p* < 0.01; *** *p* < 0.001. n.d.: not detected.

Results showed that increasing amounts of *G. officinalis* extract reduced cancer cell viability in a dose-dependent way: 50 μg/mL, 100 μg/mL and 500 μg/mL extract reduced E705 cell viability to 52%, 27% and 14%, respectively, and SW480 cell viability to 75%, 60% and 19%, respectively (Figure 2). This different sensitivity between the two cell lines was likely related to the presence of a *KRAS* activating mutation in the SW480 cells. On the contrary, CCD 841 control cells showed a very good safety profile, with their viability remaining around 85% and 80% in the presence of 50 and 100 μg/mL extract, respectively, decreasing to 65% upon the addition of 500 μg/mL extract. Moreover, increasing amounts of *G. officinalis* extract promoted apoptosis in the E705 cell line, reducing anti-apoptotic Bcl-2 expression and leading to proapoptotic caspase-3 cleavage; variations in both these markers were found to be dose-dependent (Figure 3C,D). No changes in markers related to apoptosis were observed in the healthy CCD 841 cell line (Figure 3A,B), whereas in the SW480 cell line, *G. officinalis* extract only led to a reduction in the level of Bcl-2 (Figure 3E,F). Bcl-2 downregulation is normally accompanied by caspase cleavage and apoptosis; however, since Bcl-2 also delays G_1_/S transition [49], its downregulation may facilitate progression into cell cycle, as suggested by the higher viability of SW480 cells compared to E705 ones.

**Figure 4:**
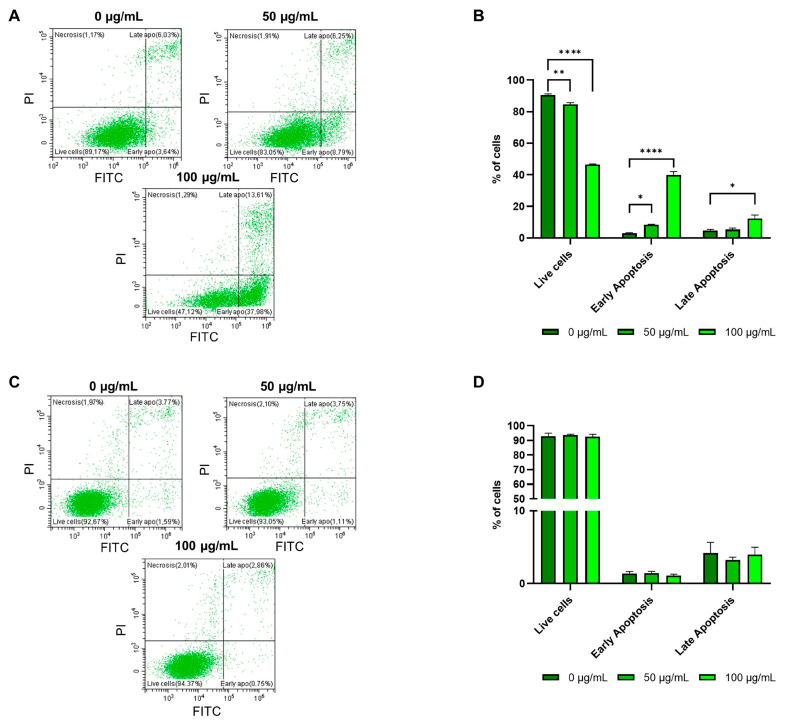
Apoptosis analysis by flow cytometry in E705 and SW480 cell lines. Representative scatter plots show the distribution of annexin V- and PI-stained cells after treatment for 24 h with *G. officinalis* extract at 50 μg/mL and 100 μg/mL with respect to the control condition (0 μg/mL) in E705 cells (**A**) and in SW480 cells (**C**). The X-axis indicates annexin V–FITC fluorescence detected at 518 nm and the Y-axis indicates PI fluorescence detected at 620 nm. The lower left quadrant indicates live cells, the upper right quadrant indicates late apoptotic cells, the lower right quadrant indicates early apoptotic cells. The results for E705 and SW480 cells are expressed as the mean percentage of total cell numbers ± standard error (SE) of three individual experiments (**B**,**D**). Statistical significance: * *p* < 0.05; ** *p* < 0.01; **** *p* < 0.0001.

The proapoptotic effect of *G. officinalis* extract was confirmed also by flow cytometry analysis, in which a dose-dependent decrease in live cells followed by a parallel increase in both early and late apoptotic cells was observed only in the E705 cancer cell line (Figure 4A,B), with no changes in the amount of live and apoptotic cells in the SW480 tumor cells (Figure 4C,D).

### 2.3. G. officinalis Extract Shows an Antiproliferative Effect on E705 Cells and Leads SW480 Cells to Cell Cycle Arrest in the G_2_/M Phase

**Figure 5:**
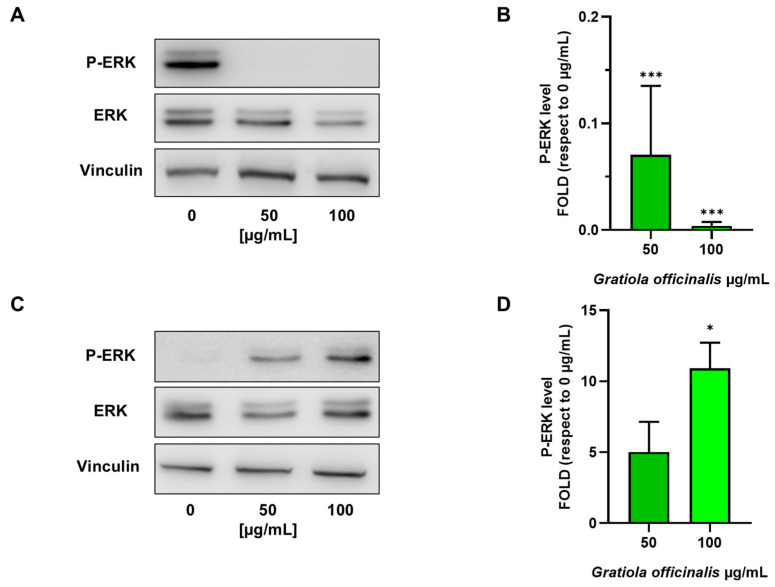
Western blot analysis. Representative Western blot analyses performed on E705 (**A**) and SW480 (**C**) cell lines that were untreated and treated for 24 h with 50 μg/mL and 100 μg/mL of *G. officinalis* extract. Protein extracts were separated on 10% acrylamide/bis-acrylamide SDS-PAGE and the nitrocellulose membranes were probed with anti-P-ERK and anti-ERK antibodies. Vinculin was used as a loading control. Densitometric analysis values for E705 and SW480 cells are expressed as folds with respect to the control condition (0 μg/mL) and are presented as the mean ± standard error (SE) of three individual experiments (**B**,**D**). Statistical significance: * *p* < 0.05; *** *p* < 0.001.

As reported in Figure 5A,B, Western blot analysis after the treatment of E705 cells with *G. officinalis* extract at 50 μg/mL and 100 μg/mL doses suggests an antiproliferative effect, promoted by the downregulation of the EGFR signaling pathway, which is normally upregulated in colorectal cancer cells [50]. In line with the proapoptotic effect, ERK phosphorylation was found to be downregulated in a dose-dependent way by *G. officinalis* extract administration. This is well in accordance with the fact that E705 cells are not KRAS-mutated and their ability to proliferate is due to the hyperactivation of the EGFR signaling pathway. On the contrary, in the SW480 cell line, *G. officinalis* extract caused a dose-dependent increase in the ERK phosphorylation level (Figure 5C,D).

**Figure 6:**
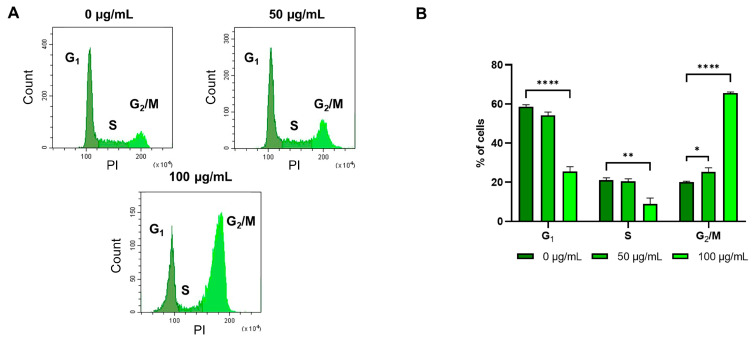
Cell cycle analysis on SW480 cell lines that were untreated and treated for 24 h with 50 μg/mL and 100 μg/mL of *G. officinalis* extract. Panel (**A**) shows the cells analyzed for DNA content by flow cytometry, while the graph in panel (**B**) depicts the percentage of cells in the different phases of the cell cycle. The values are presented as the mean ± standard error (SE) of three individual experiments. Statistical significance: * *p* < 0.05; ** *p* < 0.01; **** *p* < 0.0001.

Although this may look contradictory, since ERK phosphorylation normally triggers cell survival, other authors have observed that a persistent over-activation of ERK can induce cell cycle arrest by indirectly enhancing cdc2 phosphorylation and hampering its dephosphorylation, preventing cdc25 migration into the nucleus [51,52]. In order to assess this possibility, we conducted a cell cycle flow cytometric analysis on the SW480 cell line. The evaluation of the percentage of cells in the different cell cycle phases revealed that cells treated with *G. officinalis* extract were blocked in the G_2_/M phase; a dose-dependent decrease in the percentage of cells in the G_1_ and S phases, together with an increase in cells in the G_2_ phase, was observed (Figure 6). We could hypothesize that in *KRAS*-mutated cells, *G. officinalis* plays a role in cell cycle progression, initially enhancing the G_1_/S transition by downregulating Bcl-2 and subsequently blocking the cells in the G_2_/M phase, due to ERK hyperactivation.

### 2.4. G. officinalis Extract Promotes Glycolysis Downregulation, Reducing the Expression of Warburg Effect Markers in E705 Cells

**Figure 7:**
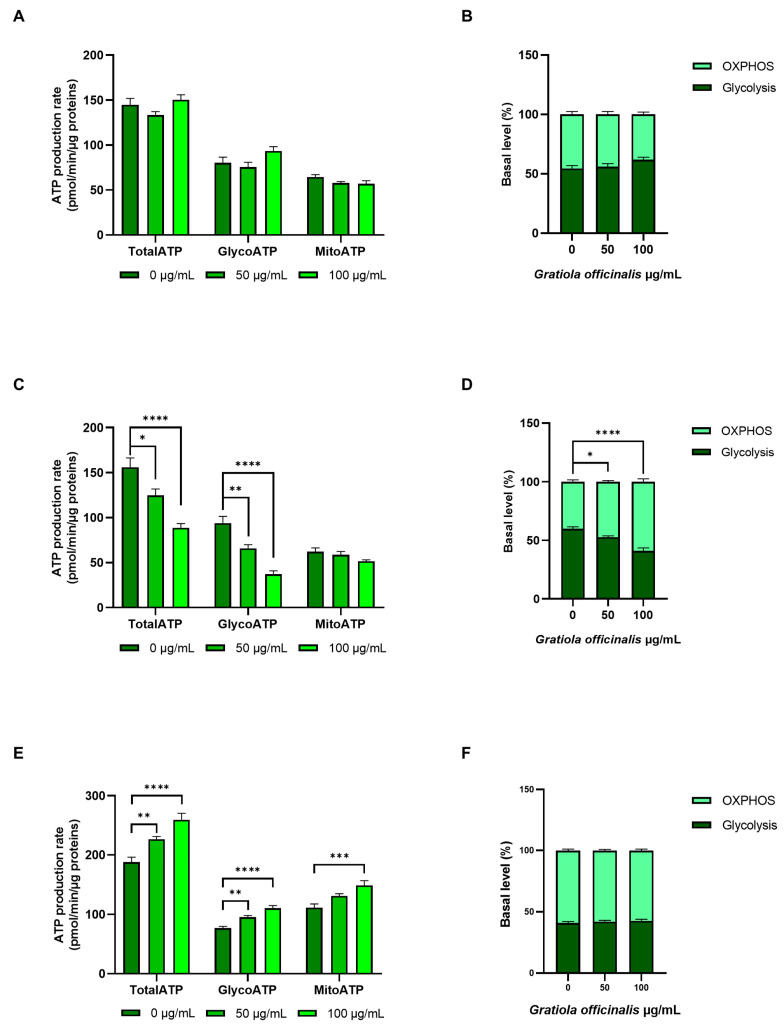
ATP production through Seahorse analysis. Total, glycolytic and mitochondrial ATP production rate in CCD 841 (**A**), E705 (**C**) and SW480 (**E**) cell lines that were untreated and treated for 24 h with 50 μg/mL and 100 μg/mL of *G. officinalis* extract. The values are presented as the mean ± standard error (SE) of three individual experiments. The ratios between glycolytic and mitochondrial ATP production in CCD 841, E705 and SW480 are reported in panels (**B**), (**D**) and (**F**), respectively. Statistical significance: * *p* < 0.05; ** *p* < 0.01; *** *p* < 0.001; **** *p* < 0.0001.

Seahorse technology was used to investigate metabolic rearrangements caused by *G. officinalis* extract administration by evaluating both the medium acidification (ECAR) and oxygen consumption (OCR) rate, measures of glycolysis and oxidative phosphorylation, respectively. Analysis of the ECAR and OCR profiles allowed the determination of total ATP production, as well as the relative contribution to ATP synthesis of the glycolytic pathway and oxidative phosphorylation. In each experiment, the basal OCR and ECAR were initially measured, with the latter being due to both lactate production through glycolysis and CO_2_ produced by oxidative phosphorylation. Subsequent oligomycin addition inhibited mitochondrial ATP synthase; this decreased the OCR and increased the glycolytic rate to balance ATP production. Following rotenone/antimycin addition, electron transport was inhibited, allowing us to measure the ECAR due to lactate production. Results, reported in Figure 7, showed no alterations in CCD 841 cells’ ATP production, following 24 h of *G. officinalis* treatment (Figure 7A,B). On the contrary, increasing *G. officinalis* extract concentrations led to a significant decrease in total ATP production in E705 cells, resulting from a reduction in glycolytic ATP, while mitochondrial ATP production remained constant (Figure 7C). Glycolysis downregulation re-equilibrated the ratio between glycolytic and mitochondrial ATP production, shifting it from a ratio of 60:40, typical of cancer cells, to a ratio of 40:60, closer to healthy cells values (Figure 7D) [53]. In SW480 cells, treatment with *G. officinalis* extract resulted in a significantly higher total ATP content, due to the increase in both glycolytic and mitochondrial ATP (Figure 7E), with no change in the ratio between glycolytic and mitochondrial ATP production (Figure 7F); this likely correlated with the initial increase in SW480 cell proliferation, triggered by Bcl-2 downregulation.

Given these results, the Glycolytic Stress Test was used to assess the glycolytic rate, capacity and reserve. Glycolytic capacity is the maximum ECAR reached when oxidative phosphorylation is blocked by the addition of oligomycin and indicates the maximum glycolytic rate of the system under stress; the glycolytic reserve is obtained by subtracting the glycolysis rate from the glycolytic capacity and is a measure of the cells’ ability to respond to the energetic demand.

**Figure 8:**
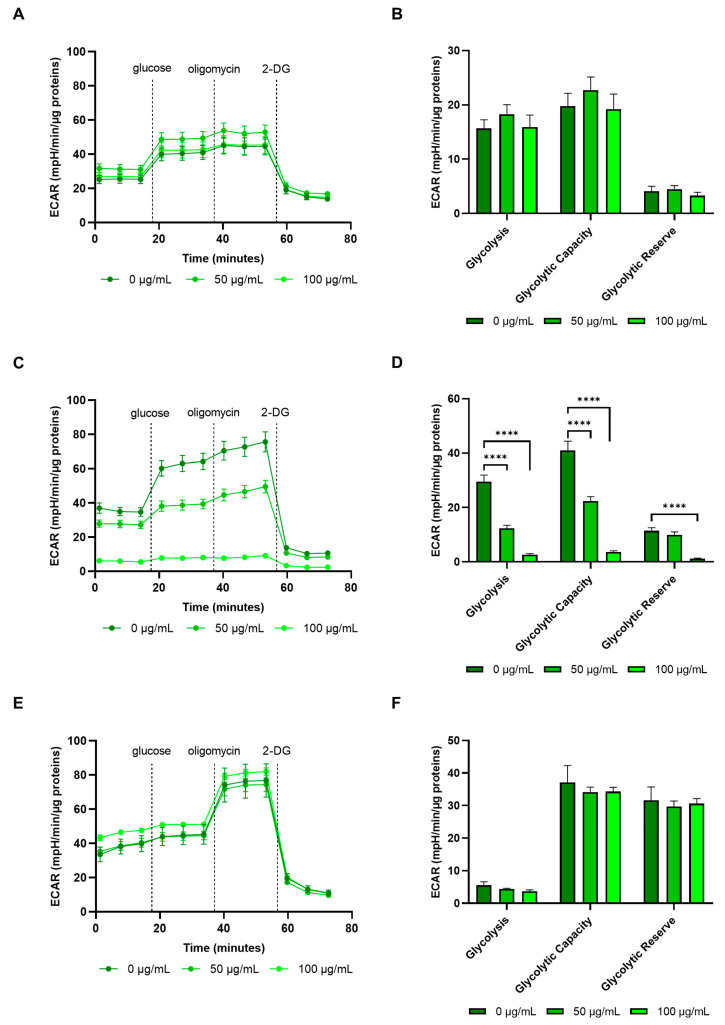
Glycolytic Stress Test. ECAR profiles, expressed as mpH/min/µg proteins, in CCD 841 (**A**), E705 (**C**) and SW480 (**E**) cell lines that were untreated and treated for 24 h with 50 μg/mL and 100 μg/mL of *G. officinalis* extract. The dotted lines indicate the time of addition of 10 mM glucose, 1 μM oligomycin and 50 mM 2-DG. The glycolysis, glycolytic capacity and glycolytic reserve parameters of CCD 841 (**B**), E705 (**D**) and SW480 (**F**) are reported. The values are presented as the mean ± standard error (SE) of three individual experiments. Statistical significance: **** *p* < 0.0001.

As in most cancer cells, the glycolytic rate, capacity and reserve of E705 and SW480 cells showed much higher values compared to healthy CCD 841 cells (Figure 8). These three parameters were all markedly reduced in the E705 cell line by *G. officinalis* extract addition, as shown in Figure 8C,D; after treatment with 50 μg/mL extract, the values of the glycolytic rate, capacity and reserve were close to the corresponding values measured in healthy CCD 841 cells, which showed no change after treatment with *G. officinalis* extract (Figure 8C,D). Treatment with 100 μg/mL extract led to an even more marked reduction in the three parameters in E705 cells, with values much lower than those found in healthy CCD 841 cells; in particular, the glycolytic reserve was completely exhausted. The fact that, at this concentration, the ECAR does not increase following oligomycin addition, which inhibits mitochondrial ATP synthase, shows that these cells already use glycolysis at its maximal rate and cannot increase ATP production; this depletion of the glycolytic reserve is what likely leads them to apoptotic death, since they rely mostly on glycolysis for ATP production.

**Figure 9:**
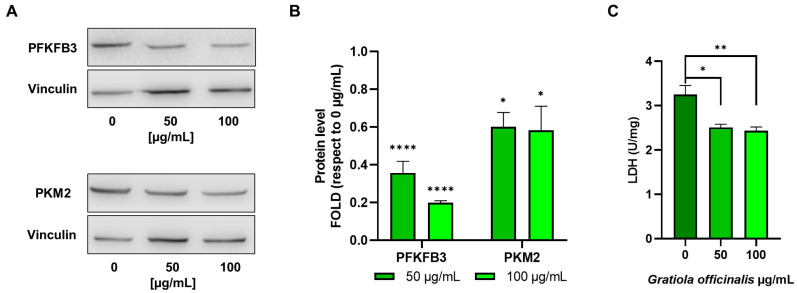
Western blot analysis. Representative Western blot analyses were performed on E705 cell lines that were untreated and treated for 24 h with 50 μg/mL and 100 μg/mL of *G. officinalis* extract. Protein extracts were separated on 10% acrylamide/bis-acrylamide SDS-PAGE and the nitrocellulose membranes were probed with anti-PFKFB3 and anti-PKM2 antibodies. Vinculin was used as a loading control (**A**). Densitometric analysis values are expressed as folds with respect to the control condition (0 μg/mL) and are presented as the mean ± standard error (SE) of three individual experiments (**B**). Panel (**C**) shows the enzymatic activity of lactate dehydrogenase, expressed in U/mg, of E705 cells that were untreated and treated for 24 h with 50 μg/mL and 100 μg/mL of *G. officinalis* extract. Bars indicate the mean ± standard error (SE) of three individual experiments. Statistical significance: * *p* < 0.05; ** *p* < 0.01; **** *p* < 0.0001.

Western blot analysis carried out on E705 after *G. officinalis* extract treatment, reported in Figure 9A,B, showed a marked decrease in the expression of phosphofructokinase 3 (PFKFB3) and pyruvate kinase 2 (PKM2). These two enzymes are isoforms of phosphofructokinase and pyruvate kinase, respectively, which are normally expressed in cancer cells and are part of the metabolic rearrangements leading to the Warburg effect [54]. In this metabolic rewiring, they promote glycolysis upregulation and lead to an increase in lactate production, which is necessary to regenerate NAD^+^ and allow glycolysis to work at sustained rates. Therefore, the downregulation of PFKFB3 and PKM2 expression is the mechanism through which *G. officinalis* extract can induce glycolysis downregulation, as demonstrated by the fact that extract addition does not change mitochondrial ATP production. Moreover, E705 cells treatment with *G. officinalis* extract led to a significant decrease in lactic dehydrogenase (LDH) activity (Figure 9C). The reduction in LDH activity is in line with the downregulation of glycolysis and lactic fermentation and suggests that *G. officinalis* extract is also effective in reducing cancer cells’ microenvironment acidity, preventing metastasis development. Conversely, in SW480 cells, the glycolytic rate, capacity and reserve values were not altered by treatment with *G. officinalis* extract (Figure 8E,F). Moreover, the increase in ECAR measured upon oligomycin addition shows that SW480 cells do not use glycolysis at the maximal rate and are therefore able to upregulate this pathway, when needed; this, in turn, leads to the Warburg effect, promoting cell survival.

## 3. Materials and Methods

### 3.1. Cell Cultures

The CCD 841 (CRL-1790™ ATCC, Manassas, VA, USA) human healthy colon mucosa cell line was grown in EMEM medium supplemented with heat-inactivated 10% fetal bovine serum (FBS), 2 mM L-glutamine, 0.1 mM non-essential amino acids, 100 U/mL penicillin and 100 µg/mL streptomycin. E705 (kindly provided by Fondazione IRCCS Istituto Nazionale dei Tumori, Milan, Italy) and SW480 (CCL-228™ ATCC, Manassas, VA, USA) human colorectal cancer cell lines were grown in RPMI 1640 medium supplemented with heat-inactivated 10% FBS, 2 mM L-glutamine, 100 U/mL penicillin and 100 µg/mL streptomycin. All cell lines were maintained at 37 °C in a humidified 5% CO_2_ incubator. Cell lines were validated by short tandem repeat profiles that were generated by the simultaneous amplification of multiple short tandem repeat loci and amelogenin (for gender identification). All the reagents for cell cultures were supplied by EuroClone (EuroClone S.p.A, Milan, Italy).

### 3.2. Plant Material and Extract Preparation

*G. officinalis* plants were purchased from the institutional nursery “Veneto Agricoltura” (Montecchio Precalcino, Vicenza, Italy) and grown in the greenhouse facility of the University of Verona until blooming occurred. Vegetative aerial organs (leaves and young stems) were sampled from two plants and pooled. The fresh material was immediately frozen in liquid nitrogen, ground to powder using an A11 basic analytical mill (IKA-Werke, Staufen, Germany) and stored at −80 °C. About 1 g of frozen powder was extracted with 10 volumes (*w*/*v*) of 100% LC-MS grade methanol (Honeywell, Seelze, Germany). The sample was vortexed for 30 s, sonicated on ice for 10 min in a 40 kHz ultrasonic bath (SOLTEC, Milano, Italy) and centrifuged at 14,000× *g* for 10 min at 4 °C. The recovered supernatant was dried with a speed-vac system (Heto-Holten; Frederiksborg, Denmark). The dried extract was re-solubilized in an equal volume of ethanol, compatible with the following cell assays, and profiled through UPLC-ESI-HRMS analysis.

### 3.3. Phytochemical Profiling of G. officinalis Extract

*G. officinalis* extract was diluted 1:20 with LC-MS-grade water (Honeywell) and passed through 0.22 µm Minisart filters (Sartorius-Stedim Biotech, Göttingen, Germany). Phytochemical profiling was performed through untargeted metabolomics by injecting 1.2 and 10 μL into the UPLC-ESI-HRMS system operating in FAST-DDA negative and positive ionization modes, with the instruments and methods previously described [55]. Three orthogonal parameters, i.e., the accurate mass (deduced from the *m*/*z* ratio and isotopic pattern), retention time, and fragmentation pattern (from the FAST-DDA analysis), were considered for the putative identification of *G. officinalis* phytochemicals and compared with a proprietary library of authentic standard compounds, with an in silico proprietary library of plant compounds and with the scientific literature and public databases (e.g., Pubchem, MoNA, MassBank, Human Metabolome Database). When no information was available, the metabolites were putatively identified in silico with Metfrag by querying the Pubchem database with the experimental MS data. The analysis in the positive ionization mode was used only to confirm the molecular ions detected in the negative ionization mode.

### 3.4. Viability Assay

The different cell lines were seeded in 96-well microtiter plates at a density of 1 × 10^4^ cells/well, cultured in complete medium and, after 24 h, treated with *G. officinalis* extract solubilized in ethanol, at concentrations between 0 and 500 μg/mL. Ethanol concentration in the wells was 0.5% in both treated and untreated cells. Then, 24 h after treatment, cell viability was investigated using the MTT-based in vitro toxicology assay kit (Merck KGaA, Darmstadt, Germany), according to manufacturer’s protocols. After 4 h for the CCD 841 and 2 h for the E705 and SW480 cell lines, formazan crystals were solubilized with 10% Triton X-100 and 0.1 N HCl in isopropanol and absorbance was measured at 570 nm using a Spectrostar Nano Microplate Reader (BMG LABTECH, Ortenberg, Germany). Cell viability was expressed as a percentage against untreated cells used as control. Viability experiments were carried out in three technical repeats per treatment for each biological replicate, and at least three independent biological replicates were performed for each cell line.

### 3.5. SDS-PAGE and Western Blotting

For Western blot analysis, cells were seeded at a density of 6 × 10^5^ cells/60 mm dish and, 24 h after seeding, were treated with *G. officinalis* extract at 50 and 100 μg/mL for 24 h. After treatment, cells rinsed with ice-cold PBS (10 mM K_2_HPO_4_, 150 mM NaCl, pH 7.2) were lysed on ice in RIPA buffer (50 mM Tris-HCl pH 7.5, 150 mM NaCl, 1% NP-40, 0.5% sodium deoxycholate, 0.1% SDS) containing 1 μM leupeptin, 2 μg/mL aprotinin, 1 μg/mL pepstatin, 1 mM PMSF and a phosphatase inhibitor cocktail (Merck KgaA, Darmstadt, Germany). Subsequently, homogenates were obtained by being passed 5 times through a blunt 20-gauge needle fitted to a syringe and then centrifuged at 15,000× *g* for 30 min. Supernatants were analyzed for protein content by a BCA protein assay [56]. SDS-PAGE and Western blotting were carried out by standard procedures [57]. The following primary antibodies, diluted at 1:1000 and purchased from Cell Signaling Technology (Danvers, MA, USA), were used: anti-Bcl-2 (#15071), anti-caspase-3 (#14220), anti-P-ERK (#4370), anti-ERK (#4695), anti-PFKFB3 (#13123) and anti-PKM2 (#4053). Loading control anti-vinculin (V9131 Merck KgaA, Darmstadt, Germany) primary antibody was used at a dilution of 1:5000. IgG HRP anti-rabbit (dilution 1:8000, #7074 Cell Signaling Technology, Danvers, MA, USA) and IgG HRP anti-mouse (dilution 1:8000, #7076 Cell Signaling Technology, Danvers, MA, USA) secondary antibodies were used. Protein levels were visualized with an ECL detection system (EuroClone S.p.A, Milan, Italy) and quantified by the densitometry of immunoblots with ImageStudio™ software version number 5.5 (LI-COR Biosciences, Lincoln, NE, USA). Three independent biological replicates were performed for each cell line.

### 3.6. Annexin V-FITC Assay for Apoptosis

E705 and SW480 cells were seeded into 24-well plates at a density of 1 × 10^5^ cells/well and, 24 h later, treated with *G. officinalis* extract at 50 and 100 μg/mL for further 24 h. After treatment, cells were harvested by trypsinization, counted and stained with annexin V/FITC and propidium iodide (PI), according to the manufacturer’s protocol (Cat V13242, Thermo Fisher Scientific, Waltham, MA, USA). Briefly, suspension containing 1 × 10^5^ cells was diluted in 100 μL annexin V binding buffer. Subsequently, 5 μL of annexin V-FITC and 1 μL of propidium iodide were added to each tube and incubated for 15 min in the dark at room temperature. Then, samples were transferred to flow cytometer tubes in a final volume of 500 μL ice-cold annexin V binding buffer, and the cells were analyzed using a flow cytometer (CytoFLEX S, Beckman Coulter Inc., Brea, CA, USA). Data were analyzed using CytExpert 2.3 Software (Beckman Coulter Inc., Brea, CA, USA). Three independent biological replicates were performed for each cell line.

### 3.7. Cell Cycle Analysis

SW480 cells were seeded into 6-well plates at a density of 2 × 10^5^ cells per well. After 24 h of incubation, the cells were treated for the following 24 h with *G. officinalis* extract at 50 and 100 μg/mL. Then, cells were harvested by trypsinization, resuspended in PBS and centrifuged at 300× *g* for 10 min. The supernatant was discarded, and the pellet incubated with 500 μL of 70% ice-cold ethanol at 4 °C for 30 min. Subsequently, after a first incubation with 100 μg/mL RNase at 37 °C for 15 min, the cells were stained with 25 μg/mL propidium iodide (PI) and dissolved in PBS containing 0.1% NP-40 in the dark for 15 min at 37 °C. Finally, 250 μL of PBS was added and the cells were analyzed by flow cytometry (CytoFLEX S, Beckman Coulter Inc., Brea, CA, USA). Data were analyzed using CytExpert 2.3 Software (Beckman Coulter Inc., Brea, CA, USA). All chemicals were purchased from Merck KgaA, Darmstadt, Germany. Three independent biological replicates were performed.

### 3.8. ATP Production and Glycolytic Rate Measurements

The measurements of the oxygen consumption rate (OCR) and extracellular acidification rate (ECAR) were performed using the Agilent Seahorse XFe96 Analyzer (Agilent Technologies Santa Clara, CA, USA) to determine the total amount of ATP produced in living cells, distinguishing between the fractions derived from oxidative phosphorylation and glycolysis and the glycolytic function.

Cells were seeded in Agilent Seahorse 96-well XF cell culture microplates at a density of 2 × 10^4^ cells per well in 180 µL of growth medium and were allowed to adhere for 24 h in a 37 °C humidified incubator with 5% CO_2_. Subsequently, the seeded cells were treated with *G. officinalis* extract at 50 and 100 μg/mL for 24 h. In addition, before running the assay, the Seahorse XF Sensor Cartridge was hydrated and calibrated with 200 µL of Seahorse XF Calibrant Solution in a non-CO_2_ 37 °C incubator to remove CO_2_ from the media that would otherwise have interfered with measurements that were pH-sensitive.

After the treatment, for the Agilent Seahorse XF ATP Rate Assay Kit, the growth medium was replaced with 180 μL/well of Seahorse XF DMEM Medium containing 1 mM pyruvate, 2 mM L-glutamine and 10 mM glucose, while for the Agilent Seahorse XF Glycolysis Stress Test Kit, the medium substitution was made with XF DMEM Medium containing 2 mM L-glutamine. Subsequently, the plate was incubated in a 37 °C non-CO_2_ incubator for 1 h, before starting the experimental procedure, and the compounds were loaded into injector ports in the sensor cartridge.

The Agilent Seahorse XF ATP Rate Assay Kit and Agilent Seahorse XF Glycolysis Stress Test Kit were used according to manufacturers’ instructions. For each biological replicate, a technical quadruplicate was performed, and data were normalized for the total protein content, quantified by the Bradford assay [58].

All the kits and reagents were purchased from Agilent Technologies (Santa Clara, CA, USA).

### 3.9. Enzyme Activity Assay

To evaluate the effect of *G. officinalis* extract on lactate dehydrogenase (LDH) activity, E705 cells were seeded at a density of 1 × 10^6^/100 mm dish and, 24 h later, treated with *G. officinalis* extract at concentrations of 50 and 100 μg/mL for 24 h. The cells were then rinsed with ice-cold PBS and lysed in 50 mM Tris-HCl with a pH of 7.5, 150 mM NaCl, 5 mM EDTA, 10% glycerol and 1% NP-40 buffer, also with 1 μM leupeptin, 2 μg/mL aprotinin, 1 μg/mL pepstatin and 1 mM PMSF. Homogenates were obtained by being passed 5 times through a blunt 20-gauge needle fitted to a syringe and then centrifuged at 15,000× *g* for 30 min. The resulting supernatant was used to measure LDH activity according to Bergmeyer [59] and the protein content was quantified by the Bradford assay [58]. All chemicals were purchased from Merck KgaA, Darmstadt, Germany. Two technical repeats were performed for each biological replicate and at least three independent biological replicates were carried out.

### 3.10. Statistical Analysis

All the experiments were carried out in biological triplicate. The samples were compared to their reference controls and the data were tested by Dunnett’s multiple comparison procedure (GraphPad Prism Software version 8.0.2). Results were considered statistically significant at *p* < 0.05.

## 4. Conclusions

Despite its long-term use in traditional medicine, *Gratiola officinalis* has not been deeply investigated so far, with regard to its biological activities. Our work shows an interesting anticancer effect of *G. officinalis* extract against colorectal cancer, a type of cancer whose rate is declining in adults but rising in young people.

In E705 cells, representative of most CRC patients, the reduction in the ability to use glycolysis and a specific downregulation of some Warburg effect markers suggest a potential role of *G. officinalis* extract in CRC prevention and therapy. In SW480 cells, representative of a less common but more aggressive cancer phenotype, the cell cycle arrest promoted by *G. officinalis* extract can also be further investigated as a coadiuvant therapeutic approach. The use of cultured cancer cells is a limitation of this work, which does not take into account the fact that polyphenols are normally metabolized by the gut microbiota; however, different data suggest that unabsorbed metabolites can act as prebiotic, while at the same time contrasting the growth of pathogenic bacteria.

Since a limited number of compounds were present in the extract, future work will regard analyzing single components for anticancer activity and specific molecular targets, establishing whether the extract activity relies on a single molecule or on the whole mixture, as in many phytotherapy treatments.

## Data Availability

The original contributions presented in this study are included in the article/Supplementary Materials. Further inquiries can be directed to the corresponding author(s).

## Supplementary Materials

The following supporting information can be downloaded at https://www.mdpi.com/article/10.3390/ijms26052220/s1. Refs [21,24,35,36,42,43,44,45,46,47,48,60,61,62] are cited in Supplementary Materials file.
