# Supplementary material for: Gratiola officinalis Alcoholic Extract Targets Warburg Effect, Apoptosis and Cell Cycle Progression in Colorectal Cancer Cell Lines
Source: Int J Mol Sci. 2025 Feb 28;26(5):2220. doi: 10.3390/ijms26052220 (PMC11900565; doi:10.3390/ijms26052220)
Supplement: Supplementary file 1 [file ijms-26-02220-s001.zip › ijms-3476469-supplementary.pdf]

SUPPLEMENTARY FILE 1: PUTATIVE IDENTIFICATIONS OF *G. officinalis* PHYTOCHEMICALS

1. Putative identifications of the phenylethanoid/phenylpropanoid glycoside esters

Peak 13 represents the highest peak of *G.officinalis* extract and likely reflects the presence of two co-eluting isomers that have been previously reported in high amounts in this and other species from the Plantaginaceae. **Arenarioside** was early isolated from the leaves of *G. officinalis* (Rothenburger & Haslinger, 1994) but, more recently, it was reported in this species also the presence of its isomer, **forsythoside B** (Šliumpaite et al., 2013). These authors applied a chromatographic method similar to the one used in our paper and reported very close retention times for these two metabolites.

The putative identity of **peak 15**, **samioside**, was attributed on the basis of the fragmentation pattern and of the elution order (following forsythoside B and verbascoside in C_{18} RP-chromatography) previously observed by (Šliumpaite et al., 2013) and also reported in other LC-MS profiling of phenylethanoid/phenylpropanoid glycosides (e.g., (Kırmızıbekmez et al., 2005). This information supported also the putative identification of **alyssonoside** (**peak16**), which, differently from the previously discussed caffeoyl-based phenylethanoid esters, presents the neutral loss of 176.04 Da and the diagnostic fragments at m/z 175.0405; 160.0168; 193.0520 that characterize its feruloyl moiety. The same pattern was found for **martynoside** (**peak 25**;(Kırmızıbekmez et al., 2005).

Echinacoside (**peak 10**) and **verbascoside** (**peak 14**) were identified by comparison with the authentic standards. Accordingly to the elution order reported by (Kırmızıbekmez et al., 2005) and to the profiling by (Šliumpaite et al., 2013), one of the two **verbascoside isomers** detected (peaks **18-19**) was putatively identified as **isoverbascoside**. Furthermore, **peak 3** exhibited similar fragmentation to verbascoside and, given the difference of 162.03 Da (-caffeoyl) between the two molecular ions and the lower retention time, this metabolite was identified as **decaffeoyl-verbascoside** (i.e. verbasoside; (Peixoto et al., 2023). **Peak 21** exhibited a molecular ion with a difference of 42.01 Da (-acetyl) with respect to that of verbascoside and showed the same fragments, thus leading to the identification as **acetyl-verbascoside**.

Peak 5 presented neutral losses of 132.04 and 146.06 Da from 593.2104 m/z indicating the presence, respectively, of pentose and deoxyhexose sugars linked, at different positions, to the core structure of the hydroxytyrsol hexoside (m/z 315.1108). This compound, whose molecular formula is $C_{25}H_{38}O_{16}$, could be either **markhamioside A or peiioside B**, which differ for the O-linkage position of the pentose residue and were both found in species from families related to Plantaginaceae such as Lamiaceae (Song et al., 2023), Bignoniaceae (Ali et al., 2015) and Verbenaceae (Wu et al., 2013). This metabolite is more likely to be markhamioside A given the larger distribution of this compound within the Lamiales.

The identity of **leucosceptoside A** (no peak id, minor compound) was attributed on the basis of its detection in other genera from the family Plantaginaceae, such as *Globularia* (Rodríguez-Pérez et al., 2019) and *Plantago* (Gao et al., 2022), and on the match with the fragmentation pattern reported by the authors.

2. Putative identifications of the cucurbitane-type triterpenoids

Cucurbitacins derivatives:

Cucurbitacins B (**peak 45**)**, E** (**peak 44**)**, I, S** and their **2-O glucosides** (**peaks 27, 34, 36, 37**) were identified by comparison with the fragmentation data reported by (Ul Haq et al., 2019) and (Hu et al., 2023). To our knowledge, this is the first work reporting the presence of Cucurbitacin S and its 2-O-glucoside in *G. officinalis*.

Gratiogenin derivatives:

Glycosides of **gratiogenin** (**peaks 32, 38, 39**) and **16-hydroxygratiogenin** (peaks 28, 35) were putatively identified on the basis of the information reported by (Rothenburger & Haslinger, 1995) and of the neutral losses of 132.04 (pentosyl) and 162.05 (hexosyl) Da observed, leading to the detection of the two genins (471.3474 m/z for gratiogenin 487.3423 m/z for 16-hydroxygratiogenin). The metabolites for which we have specified the sugar residue (e.g., apiose or glucose) and the corresponding bond position on the aglycone structure have been previously described in *G. officinalis* by (Rothenburger & Haslinger, 1995).

Furthermore, metabolites of peaks 32, 38, 39 were also detected in their **acetylated+carboxylated derivatives** (**peaks 33, 40, 41**) and **diacetylated+carboxylated derivative** (**peak 43**). All these compounds produced a fragmentation pattern in which are observed the neutral losses of 43.99 Da (CO_2) and 42.01 Da (CH_3CO), typical of -carboxyl and -acetyl residues, followed by the neutral losses of the respective sugar moieties, leading to the detection of the gratiogenin ion (m/z 471.3524). This structural modification shifts the elution of these metabolites to higher retention times.

Other terpenoids:

Peak 31 showed consecutive neutral losses of 132.04 Da (pentosyl) and 162.04 Da (hexosyl) leading to an ion with m/z 489.3596. Given the high number of isomers corresponding to the genin with the formula $C_{30}H_{50}O_5$, the identity of this metabolite was not assigned. Similarly, **peak 29** and **peak 42** were annotated as the O-hexoside and di-O-hexoside, respectively, of putative triterpenoids with the formula $C_{30}H_{46}O_6$ and $C_{30}H_{44}O_5$. The latter was detected also as its acetylated+carboxylated derivative.

Betulinic acid (peak 46) was previously reported in *G. officinalis* (Ali et al., 2012) and identified by comparison with authentic standard.

3. Putative identifications of other compounds

Rt 2.82 min – Dihydroxybenzoic acid sulfohexoside

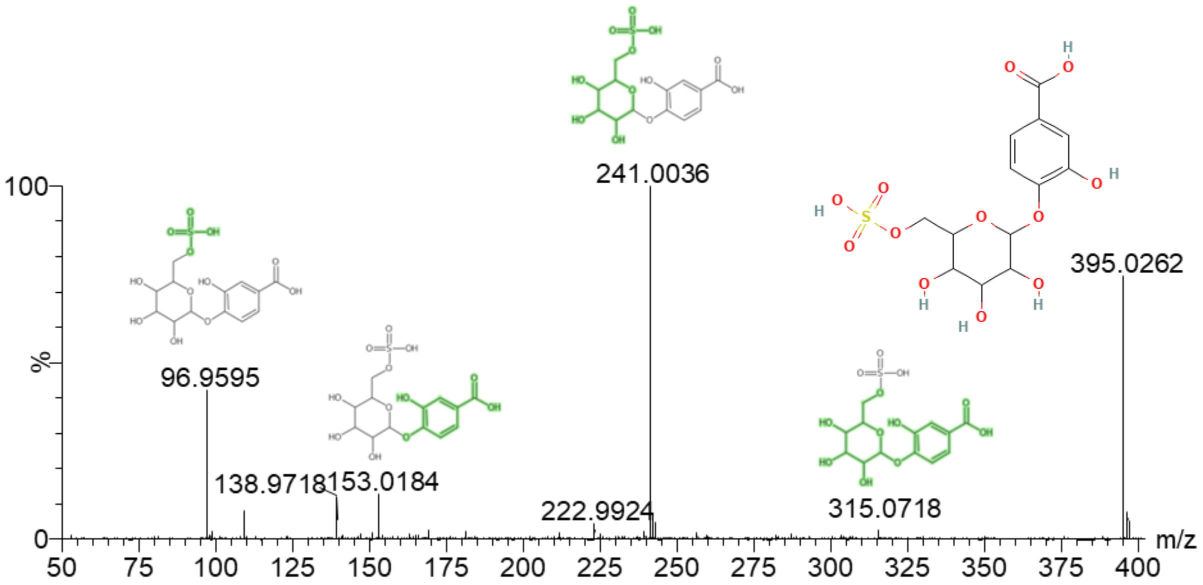

This compound was putatively identified through in-silico fragmentation with MetFrag (https://msbi.ipb-halle.de/MetFrag/) by querying the m/z of the molecular ion detected (395.0262), the calculated molecular formula ($C_{13}H_{16}O_{12}S$) and the observed fragmentation pattern against the Puchem database.

Peak 4: 2,5-dihydroxy-*p*-benzendiacetic acid

The fragmentation of this compound highlights neutral losses of 43.99, 28.00 and 18.01 Da attributed to the presence of various -COOH and -OH residues. This pattern was previously observed in *G. officinalis* by (Šliumpaite et al., 2013), who putatively identified this compound as 2,3-dihydroxybenzendiacetic acid.

Rt 4.64 min – Caffeic acid glycoside

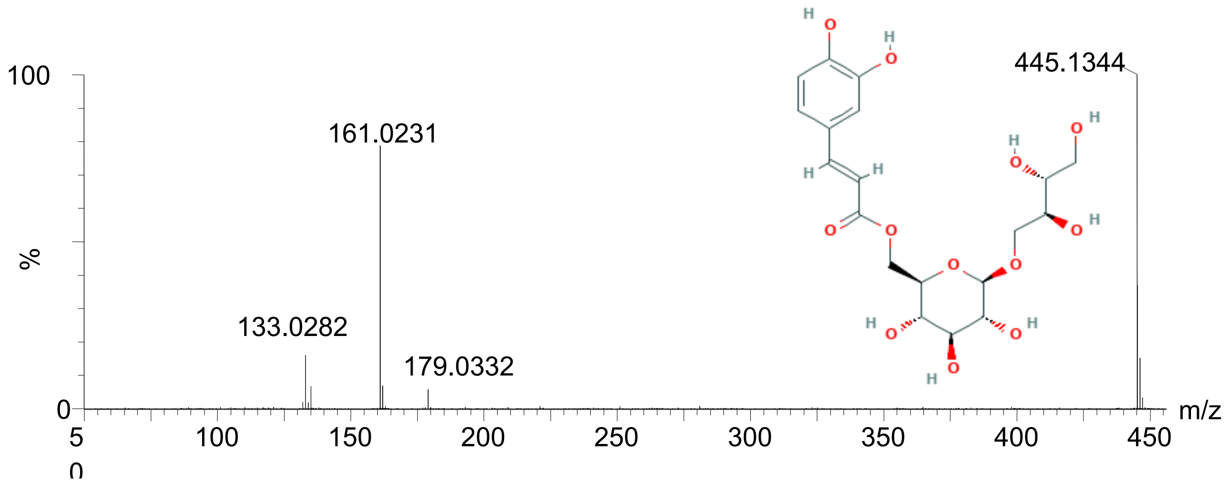

The fragmentation of this metabolite suggests the presence of a caffeoyl moiety. It was performed the in-silico retrieval with MetFrag by querying the m/z of the molecular ion detected (445.1344), the calculated molecular formula ($C_{19}H_{26}O_{12}$) and the observed fragmentation pattern against the Puchem database. The output with the highest score was the following caffeic acid glycoside: [(2R,3S,4S,5R,6R)-3,4,5-trihydroxy-6-[(2S,3R)-2,3,4-trihydroxybutoxy]oxan-2-yl]methyl (E)-3-(3,4-dihydroxyphenyl)prop-2-enoate.

Rt 7.82 min – Dihydrodehydrodiconiferyl alcohol-9'-O-sulfate (peak 24)

This neolignan sulfate was putatively identified through in silico retrieval with MetFrag by querying the m/z of the molecular ion detected (439.1071), the calculated molecular formula ($C_{20}H_{24}O_9S$) and the observed fragmentation pattern against the Puchem database. The two fragments at m/z 96.9695 and 79.9566 were diagnostic of the presence of a sulfate group. This metabolite was previously identified through NMR in *Glochidion zeylanicum* (Euphorbiaceae; (OTSUKA et al., 2000).

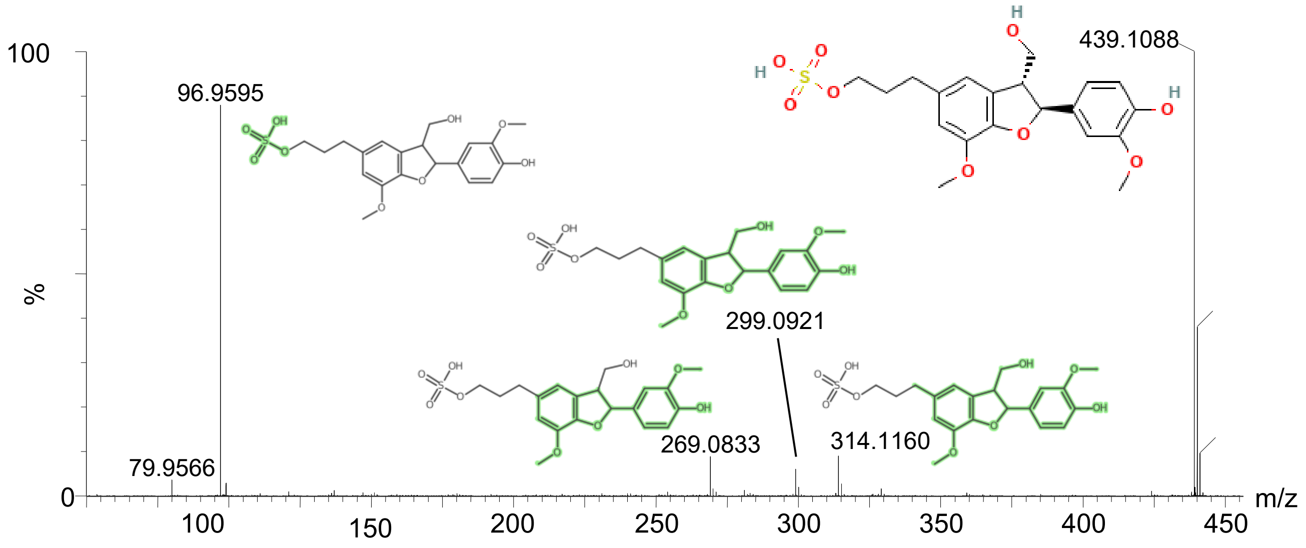

References

Ali, L., Rizvi, T. S., Ahmad, M., & Shaheen, F. (2012). New iridoid glycoside from *Gratiola officinalis*. *Journal of Asian Natural Products Research*, *14*(12), 1191–1195. https://doi.org/10.1080/10286020.2012.734299

Ali, S., El-Ahmady, S., Ayoub, N., & Singab, A. (2015). Phytochemicals of Markhamia Species (Bignoniaceae) and Their Therapeutic Value: A Review. *European Journal of Medicinal Plants*, 6(3), 124–142. https://doi.org/10.9734/EJMP/2015/15015

Gao, H., Liu, Z., Song, F., Xing, J., Zheng, Z., & Liu, S. (2022). A Strategy for Identification and Structural Characterization of Compounds from Plantago asiatica L. by Liquid Chromatography-Mass Spectrometry Combined with Ion Mobility Spectrometry. *Molecules*, *27*(13), 4302. https://doi.org/10.3390/molecules27134302

Hu, G., Liu, W., & Li, L. (2023). Identification and quantification of cucurbitacin in watermelon frost using molecular networking integrated with ultra-high-performance liquid chromatography-tandem mass spectrometry. *Journal of Separation Science*, *46*(16). https://doi.org/10.1002/jssc.202300019

Kırmızıbekmez, H., Montoro, P., Piacente, S., Pizza, C., Dönmez, A., & Çalış, İ. (2005). Identification by HPLC-PAD-MS and quantification by HPLC-PAD of phenylethanoid glycosides of five *Phlomis* species. *Phytochemical Analysis*, *16*(1), 1–6. https://doi.org/10.1002/pca.802

OTSUKA, H., HIRATA, E., SHINZATO, T., & TAKEDA, Y. (2000). Isolation of Lignan Glucosides and Neolignan Sulfate from the Leaves of Glochidion zeylanicum (Gaertn) A. Juss. *Chemical and Pharmaceutical Bulletin*, *48*(7), 1084–1086. https://doi.org/10.1248/cpb.48.1084

Peixoto, J. A. B., Álvarez-Rivera, G., Costa, A. S. G., Machado, S., Cifuentes, A., Ibáñez, E., Oliveira, M. B. P. P., & Alves, R. C. (2023). Contribution of Phenolics and Free Amino Acids on the Antioxidant Profile of Commercial Lemon Verbena Infusions. *Antioxidants*, *12*(2), 251. https://doi.org/10.3390/antiox12020251

Rodríguez-Pérez, C., Zengin, G., Segura-Carretero, A., Lobine, D., & Mahomoodally, M. F. (2019). Chemical fingerprint and bioactivity evaluation of *Globularia orientalis* L. and *Globularia trichosantha* Fisch. & C. A. Mey. using non-targeted HPLC-ESI-QTOF-MS approach. *Phytochemical Analysis*, *30*(2), 237–252. https://doi.org/10.1002/pca.2809

Rothenburger, J., & Haslinger, E. (1994). Caffeic Acid Glycoside Esters from *Gratiola officinalis* L. *Liebigs Annalen Der Chemie*, *1994*(11), 1113–1115. https://doi.org/10.1002/jlac.199419941110

Rothenburger, J., & Haslinger, E. (1995). New cucurbitacine glycosides fromGratiola officinalis L. *Monatshefte Für Chemie - Chemical Monthly*, *126*(12), 1331–1339. https://doi.org/10.1007/BF00807062

Šliumpaite, I., Venskutonis, P. R., Murkovic, M., & Pukalskas, A. (2013). Antioxidant properties and polyphenolics composition of common hedge hyssop (Gratiola officinalis L.). *Journal of Functional Foods*, *5*(4), 1927–1937. https://doi.org/10.1016/J.JFF.2013.09.014

Song, J., Liu, Y., Yin, X., Nan, Y., Shi, Y., Chen, X., Liang, H., Zhang, J., & Ma, B. (2023). Isolation and structural elucidation of prebiotic oligosaccharides from Ziziphi Spinosae Semen. *Carbohydrate Research*, *534*, 108948. https://doi.org/10.1016/J.CARRES.2023.108948

Ul Haq, F., Ali, A., Khan, M. N., Shah, S. M. Z., Kandel, R. C., Aziz, N., Adhikari, A., Choudhary, M. I., ur-Rahman, A.-, El-Seedi, H. R., & Musharraf, S. G. (2019). Metabolite Profiling and Quantitation of Cucurbitacins in Cucurbitaceae Plants by Liquid Chromatography coupled to Tandem Mass Spectrometry. *Scientific Reports*, *9*(1), 15992. https://doi.org/10.1038/s41598-019-52404-1

Wu, A.-Z., Zhai, Y.-J., Zhao, Z.-X., Zhang, C.-X., Lin, C.-Z., & Zhu, C.-C. (2013). Phenylethanoid glycosides from the stems of Callicarpa peii (hemostatic drug). *Fitoterapia*, *84*, 237–241. https://doi.org/10.1016/j.fitote.2012.12.014
